# Experimental Evaluation of the Mechanical Healing Performance of Precast Concrete Incorporating Hybrid Capsules Under Load Reapplication for Smart Construction Material

**DOI:** 10.3390/ma19102003

**Published:** 2026-05-12

**Authors:** Yong Jic Kim, Sung-Rok Oh, Myounghwi Kim, Hyung-Suk Kim

**Affiliations:** 1 Department of Smart City Construction Convergence Engineering, Daejin University, Pocheon 11159, Republic of Korea; yong1yong2@hotmail.com (Y.J.K.); myounghwi.kim@daewooenc.com (M.K.); 2 R&D Team, Newjust Co., Ltd., Gwangmyeong 14348, Republic of Korea; 3 R&D Team, Quotient Time Co., Ltd., Namyangju 12106, Republic of Korea

**Keywords:** hybrid capsules, precast concrete, self-healing concrete, mechanical healing performance, durability correlation, smart construction applications

## Abstract

This study experimentally evaluates the mechanical healing performance of precast concrete incorporating hybrid capsules under load reapplication conditions. Hybrid capsule systems are defined as self-healing systems that combine solid capsules (SCs) and liquid capsules (LCs), to enable multi-scale crack healing. In this study, four mix proportions (HC-0, HC-1, HC-3, and HC-5), corresponding to 0%, 1%, 3%, and 5% replacement of fine aggregate by volume with hybrid capsules, were prepared. The hybrid capsules consisted of SCs and LCs in a fixed ratio of 7:3. Among the mixtures, a representative intermediate content (3%) was selected to examine the feasibility of mechanical recovery compared to plain concrete, rather than to determine an optimal dosage. Mechanical recovery was evaluated through compressive and flexural strength tests after preloading and healing periods. The results confirm that the incorporation of hybrid capsules enables partial recovery of mechanical properties after damage. These findings provide preliminary experimental evidence of the feasibility of hybrid capsule systems in precast concrete. Further studies are required to investigate the influence of capsule content and to establish optimal mixture conditions.

## 1. Introduction

Reinforced concrete (RC) structures constructed using traditional on-site casting methods often face challenges in quality control due to weather conditions, variability in material quality, and inconsistencies in formwork installation and pouring procedures. Such factors lead to issues in structural performance and safety, resulting in increased construction costs and extended project durations [1,2]. In contrast, the precast concrete (PC) method allows major structural components to be prefabricated and cured under controlled factory conditions before being assembled on-site, thereby improving construction efficiency, quality consistency, and safety [3,4,5,6]. Consequently, precast concrete has been widely adopted in modern infrastructure, particularly for underground, sewage, and water treatment structures where rapid installation and long-term durability are critical. However, these structures are difficult to inspect or maintain after installation because of their limited accessibility. As a result, repair and replacement costs rise significantly over time [7,8].

Cracks in concrete structures occur due to various mechanical and environmental causes such as design defects, thermal and shrinkage stresses, or chemical deterioration (e.g., chloride attack, carbonation, or alkali–silica reaction) [9,10,11]. These cracks compromise durability and service life, making efficient maintenance a key engineering priority. The concept of self-healing concrete has emerged as a sustainable alternative to conventional repair strategies, enabling cracks to be autonomously sealed without human intervention. This self-restorative capability contributes to the resilience of concrete structures by reducing maintenance frequency and extending service life [10,11]. Among various self-healing approaches, capsule-based systems have drawn significant attention due to their simplicity, long-term stability, and ability to store healing agents until triggered by cracking [12,13].

Self-healing capsules are typically categorized into two groups: solid-based capsules containing powdered expansive materials, and liquid-based microcapsules containing silicate or polymeric agents. Each type has inherent advantages and drawbacks. Solid-based capsules promote healing through the formation of crystalline products such as ettringite or calcium carbonate, whereas microcapsules activate silicate-based gel formation to fill microcracks. However, solid capsules often struggle to reach finer cracks due to their larger particle size, while microcapsules, though effective for microcracks, exhibit limited performance in larger cracks. To address these limitations, hybrid capsules—a combination of solid and liquid capsules—have been introduced to achieve complementary healing reactions [13,14,15]. The solid capsule component generates calcium hydroxide, which accelerates the silicate reaction of the microcapsules, while the silicate products, in turn, stimulate further crystallization and hydration, thereby enhancing overall healing efficiency [16].

In recent years, several studies have investigated hybrid capsule systems and their influence on the healing behavior of cementitious materials [17,18]. Previous studies on capsule-based self-healing systems can be broadly classified into three categories. First, many studies have focused on mortar-scale specimens to investigate fundamental healing mechanisms, primarily through permeability recovery and crack closure under static conditions. While these studies have provided valuable insights into the chemical and microstructural aspects of healing, their applicability to structural concrete remains limited.

Second, some studies have extended the investigation to concrete-scale specimens; however, most of these studies have been conducted under unloaded or static crack conditions. As a result, the mechanical behavior of healed concrete under load reapplication, which more closely represents actual service conditions, has not been sufficiently addressed.

Third, several studies have examined either durability-related healing performance or mechanical recovery independently. However, the relationship between these two aspects has not been clearly established, resulting in a lack of integrated understanding of healing performance in cementitious materials.

Compared to previous studies, this study differs in three key aspects. First, precast concrete is used instead of mortar-scale specimens to better reflect structural applications. Second, mechanical healing performance is evaluated under controlled load reapplication levels (30%, 60%, and 80%). Third, the relationship between durability-related healing indicators and mechanical recovery is explored. Therefore, this study aims to provide a more application-oriented experimental framework for assessing the feasibility of hybrid capsule systems in structural concrete.

From a practical perspective, precast concrete elements are required to maintain structural performance even after experiencing partial damage under service loads. Therefore, this study focuses on experimentally evaluating whether hybrid capsule systems can contribute to mechanical recovery under such conditions, rather than establishing optimal mixture proportions.

In this study, the hybrid capsule system was incorporated into precast concrete to evaluate its mechanical healing performance under load reapplication. The system, consisting of solid capsules (SCs) and liquid capsules (LCs), was designed to enable multi-scale crack healing through complementary mechanisms. A representative capsule content was adopted to examine the feasibility of mechanical recovery compared to plain concrete, rather than to determine the optimal dosage. Mechanical recovery was assessed under different preloading levels (30%, 60%, and 80%), and its relationship with durability-related indicators was also examined. In the context of advanced construction materials, this study focuses on the experimental verification of autonomous recovery capability as a fundamental material property. The findings of this study provide experimentally validated insights into the potential of hybrid capsule-incorporated precast concrete for structural applications.

This study is structured as follows. Section 2 describes the materials, mixture proportions, and experimental methods, including the evaluation procedures for mechanical healing under load reapplication. Section 3 presents the experimental results and discusses the effects of hybrid capsules on mechanical recovery and durability-related healing performance. Finally, Section 4 summarizes the main findings and provides concluding remarks.

## 2. Experiment Outline

### 2.1. Hybrid Capsules

Hybrid capsules (HCs) were designed to compensate for the individual limitations of solid-phase capsules (SCs) and liquid-phase capsules (LCs) by combining both types to achieve complementary healing mechanisms and a broader particle size distribution. Solid capsules (SCs) are generally effective in promoting healing in relatively larger cracks through the formation of crystalline products, whereas liquid-phase capsules (LCs) are more suitable for filling microcracks via silicate-based reactions.

In this study, the hybrid capsule system was composed of SC and LC at a fixed ratio of 7:3 by mass. The HC content used in the mixture represents the total amount of combined capsules, while the internal proportion between SC and LC was kept constant.

**Figure 1:**
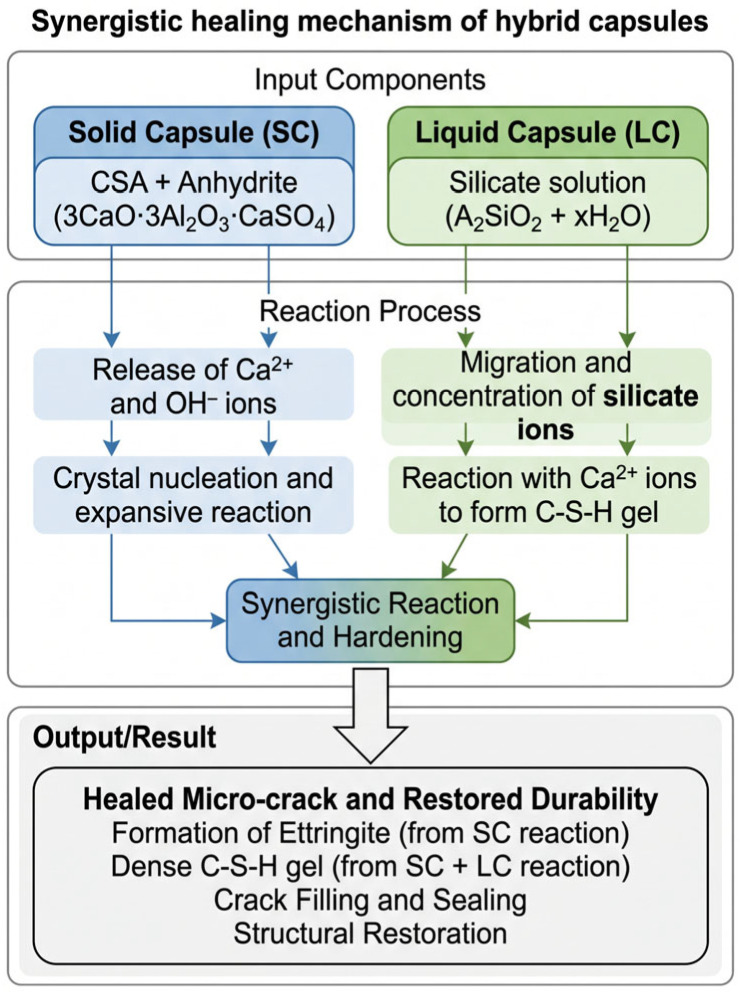
Proposed synergistic self-healing mechanism of hybrid capsules composed of solid capsules (SCs) and liquid capsules (LCs), leading to crack sealing, densification, and mechanical recovery.

In the hybrid mechanism, calcium hydroxide released from SC is expected to promote the silicate reactions of LC, while the reaction products from LC contribute to matrix densification and may enhance further hydration and crystallization around crack regions [12,13,14,15]. The conceptual mechanism of hybrid capsules (HC) is illustrated in Figure 1.

#### 2.1.1. Solid Capsules (SCs)

Solid capsules (SCs) were designed as expansive-type self-healing agents, and their cores consisted of inorganic powders primarily based on calcium sulfoaluminate (CSA; expansive agent, Denka Co., Ltd., Tokyo, Japan) and anhydrous gypsum (CaSO_4_, Hanil Cement Co., Ltd., Seoul, Republic of Korea). Upon contact with moisture, CSA forms ettringite crystals that induce expansion, while CaSO_4_ contributes to crystal growth, which may help fill crack voids [12]. Table 1 presents the chemical composition of the expansive materials.

To prepare the core, powdered raw materials were agglomerated using a low-moisture urethane-based binder to prevent premature hydration and to ensure sufficient viscosity for granulation. The granulated cores were subsequently coated with a polyurethane (Sigma-Aldrich, St. Louis, MO, USA) membrane (viscosity adjusted using toluene (Sigma-Aldrich, St. Louis, MO, USA)) to minimize premature interaction with mixing water and to enhance mechanical stability during mixing.

Following size classification, SCs with a nominal particle size of 850 μm (within the manufactured range of 600–2360 μm) was selected based on previous studies to achieve a balance between crack-bridging capability and dispersion within the matrix [12,13].

**Figure 2:**
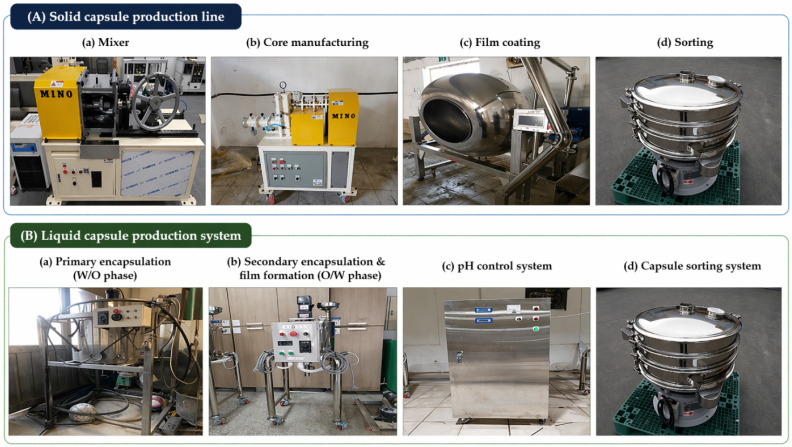
Equipment used for the fabrication of hybrid capsules. (**A**) Solid capsule production line: (**a**) mixing of shell materials, (**b**) core manufacturing, (**c**) film coating, and (**d**) sorting of solid capsules. (**B**) Liquid capsule production system: (**a**) primary encapsulation (W/O phase), (**b**) secondary encapsulation and interfacial film formation (O/W phase), (**c**) pH control, and (**d**) capsule sorting. This figure was reorganized using photographs from our previous studies [19].

**Figure 3:**
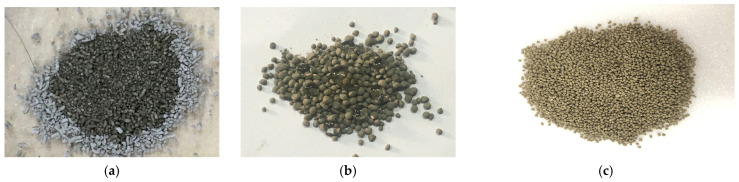
Microscope images of solid capsules (×100): (**a**) before coating (water absorption state), (**b**) after coating showing water repellency (water droplets formed on the surface), and (**c**) solid capsules with a representative particle size of 850 μm used in the experiments [20].

In the hybrid capsule system, SCs are intended to contribute primarily to the healing of relatively larger cracks through expansive and crystallization-based mechanisms, complementing the role of liquid-phase capsules (LCs) in finer crack regions. The overall SCs manufacturing process (mixing → granulation → coating → sorting) follows the procedure described in Section 2.1 of the original manuscript and Figure 2(Aa–Ad). Figure 3 presents microscope images of the solid capsules before coating, after coating, and after particle size classification.

#### 2.1.2. Liquid Capsules (LCs)

Liquid-phase capsules (LCs) were designed as silicate-based capsules, and their cores comprised inorganic silicate reactants—potassium silicate, sodium silicate, and lithium silicate (Youngil Chemical Co., Ltd., Incheon, Republic of Korea)—encapsulated within a polyurethane/urea/formaldehyde shell (Sigma-Aldrich, St. Louis, MO, USA). These silicates can react with Ca(OH)_2_ in the cement matrix to form calcium silicate hydrate (C–S–H) gels, which may contribute to sealing microcracks and densifying the surrounding microstructure. In addition, alkaline ions (K^+^, Na^+^, Li^+^) may promote further hydration of unreacted cement particles [12]. Table 2 presents the chemical composition of the silicate-based materials used in the LC core.

The viscosity of the shell solution was adjusted using toluene (target dissolution ≈ 60%) to stabilize capsule formation. After synthesis and shell formation, pH was controlled, and particles were classified to obtain a working size range of 160–350 μm from the manufactured 30–350 μm distribution, based on previous studies targeting microcrack scales and uniform dispersion [12]. The LC manufacturing process corresponds to that illustrated in Figure 2(Ba–Bd) of the original manuscript.

In the hybrid capsule system, LC are intended to contribute primarily to the healing of microcracks through silicate-based reactions, complementing the role of solid capsules (SCs) in larger crack regions.

Liquid capsules (LCs) based on silicate inorganic materials were synthesized via an in situ polymerization process using a water–oil–water (W/O/W) phase system. First, a primary encapsulation was performed in a W/O phase, where the core material was encapsulated using polyurethane (PU) through a microdroplet formation process (Figure 2(Ba). Subsequently, a secondary encapsulation was carried out in an O/W phase by mixing the primary capsules with an aqueous urea solution to form an emulsion, followed by the addition of formaldehyde to form the capsule shell (Figure 2(Bb).

**Figure 4:**
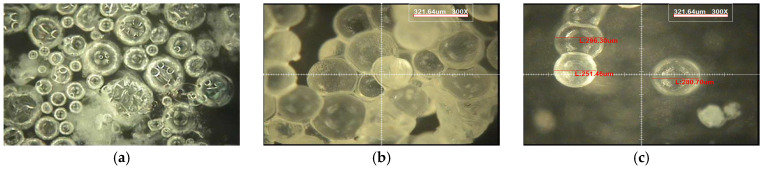
Microscope images of liquid capsules at different observation conditions (×300): (**a**) overall distribution of capsules, (**b**) enlarged view of representative capsules, and (**c**) diameter measurement of selected capsules.

To enhance the mechanical stability of the capsule shell, resorcinol was added as a reinforcing agent. The pH of the reaction system was controlled using sodium hydroxide (NaOH) and hydrochloric acid (HCl), as shown in Figure 4c. In addition, 1-octanol was used as a defoaming agent to eliminate residual and micro air bubbles during the encapsulation process.

Finally, to further strengthen the capsule shell, a silica coating was applied by polymerizing the LC in a tetraethyl orthosilicate (TEOS) aqueous solution. Monobasic sodium phosphate and tetrabutylammonium fluoride were used as auxiliary additives in this process. The synthesized capsules were then sorted to obtain uniform particle sizes (Figure 2(Bd)).

The silicate-based core material was prepared based on a composition ratio of K_2_SiO_3_:Na_2_SiO_3_:Li_2_SiO_3_ = 5:4:1, determined from previous studies.

### 2.2. Materials and Mix Proportions

Ordinary Portland cement (Type I), river sand (fine aggregate), and crushed gravel (20 mm; coarse aggregate) were used in this study. A polycarboxylate-based high-range water-reducing admixture (Eugene Admixture Co., Ltd., Jincheon, Republic of Korea), was incorporated to maintain workability and to promote uniform dispersion of capsules within the matrix. The mix design followed the KCS 14 20 52 Precast Concrete (2021) specification [21] and, where not specified, KCS 14 20 10 General Concrete (2021) [22]. The design criteria were selected to reflect typical precast concrete applications such as water-treatment structures.

The target mixture performance was established to simulate typical precast concrete for water-treatment structures in accordance with practical ranges suggested by KCS standards and field practice. A design compressive strength of 45 MPa was adopted for structural performance, whereas a slump of 170 mm and an air content of 3% were selected to achieve appropriate workability and durability. In addition, the nominal maximum size of coarse aggregate was set to 25 mm for standard precast production conditions.

Based on these target performance criteria, the mix proportions shown in Table 3 were established using a conventional mix design approach. To evaluate the effect of hybrid capsules, a portion of fine aggregate was replaced by hybrid capsules on a volume basis, while all other mixture parameters were kept constant. This approach enables a direct comparison of healing performance by minimizing the influence of other variables.

Four mixtures were prepared by replacing a portion of fine aggregate with hybrid capsules (HC) at 0%, 1%, 3%, and 5%, denoted as HC-0, HC-1, HC-3, and HC-5, respectively. The HC content represents the total amount of combined capsules, while the internal composition of HC was fixed at a ratio of 7:3 (SC:LC) as described in Section 2.1.

In this study, the selected HC contents were not intended to determine an optimal dosage, but rather to examine the feasibility of mechanical healing performance compared to plain concrete. In particular, the intermediate level (3%) was considered as a representative content to evaluate the potential effect of hybrid capsules under load reapplication conditions.

For the healing performance evaluation, mortar specimens were prepared based on the actual concrete mixture used in this study, excluding only the coarse aggregates. This approach was adopted to maintain consistency in binder composition and mixture design with the target precast concrete system while enabling controlled crack and healing assessment.

The interference of coarse aggregates refers to their influence on crack formation and propagation due to their heterogeneous distribution. Cracks may propagate irregularly along the interfacial transition zones, resulting in variability in crack geometry and connectivity. To ensure consistent and reproducible conditions for evaluating healing performance, coarse aggregates were excluded.

The mixture design was established based on the same reference matrix used in our previous study [19], with identical replacement ratios for comparative evaluation of healing performance.

### 2.3. Evaluation Methods

#### 2.3.1. Basic Quality

Slump and air content were measured in accordance with KS F 2402 and KS F 2421 [23,24]. Compressive strength was determined per KS F 2405 [25] at the specified ages. These tests were conducted to confirm that the incorporation of hybrid capsules does not adversely affect the fundamental fresh and mechanical properties of the concrete mixture.

All tests were performed under standard laboratory conditions consistent with the cited standards.

#### 2.3.2. Durability 

Durability performance was examined only to ensure that the inclusion of hybrid capsules did not compromise the fundamental durability of the precast concrete. These evaluations were not intended to provide a comprehensive durability assessment, but rather to verify the baseline compatibility of the hybrid capsule system with conventional durability requirements. Representative tests, including chloride ion diffusion, carbonation, and freeze–thaw resistance, were conducted in accordance with KS F and ASTM standards [26,27,28,29,30]. The specific procedures followed standard accelerated methods under controlled temperature, humidity, and CO_2_ concentration conditions.

#### 2.3.3. Healing Performance

Mechanical healing was assessed on 40 × 40 × 160 mm specimens molded and water-cured per KS L ISO 679 [31]. Preloading and reloading were conducted in compression and flexure to quantify strength recovery after a healing period.

(a) Definition of preloading levels and damage induction: Preloading levels were set at 0%, 30%, 60%, and 80% of the 28-day failure strength, *Su*. Here, *Su* was determined from companion specimens (same batch, same curing) tested under monotonic loading at 28 days to establish the reference failure strength for each loading mode (compressive or flexural). The induced damage level in a test specimen therefore corresponds to a prescribed fraction of *Su* in the same mode:

(1) $$Preloading\;level=\{0,\;0.30,\;0.60,\;0.80\}\times S_{u}$$

Preloading was applied under displacement control up to the target fraction to ensure consistency near the elastic (30%), inelastic (60%), and near-failure (80%) regimes, followed by unloading to zero. During preloading, visible crack initiation and propagation were monitored, particularly in flexural specimens, to confirm the level of induced damage corresponding to each preloading condition.

(b) Healing protocol: After preloading, specimens were water-cured (20 ± 2 °C) during the healing period. Reloading tests were performed at 7 days and 28 days after preloading to quantify recovery. Control specimens without preloading were also tested at corresponding ages to account for continued hydration effects and to provide an appropriate reference for strength comparison.

(c) Strength metrics and healing-rate calculation: Let $S_{\mathrm{initial}}$ denote the reference strength of the mixture at the test age (from un-preloaded control specimens), $S_{\mathrm{damaged}}$ the residual strength immediately after preloading (unloaded state), and $S_{\mathrm{recovered}}$ the measured strength upon reloading after the healing period (same loading mode). The healing rate was computed as

(2) $$\mathrm{Healing}\;\mathrm{rate}\;(\%)=\frac{S_{\mathrm{recovered}}-S_{\mathrm{damaged}}}{S_{\mathrm{initial}}-S_{\mathrm{damaged}}}\times 100$$

This definition normalizes recovered capacity against the recoverable margin, enabling comparison across mixtures and preloading levels. This approach was adopted to distinguish the effect of self-healing from the strength gain due to ongoing hydration.

For flexural tests where no measurable healing occurred in HC-0 at 80% preloading (as observed in the results), the HC-3 healing rate at the same condition is interpreted as a relative gain over HC-0 at that level.

It should be noted that the reference strength is based on 28-day specimens, and age-matched control specimens at later ages were not included; therefore, the contribution of continued hydration to strength recovery cannot be fully isolated.

**Figure 5:**
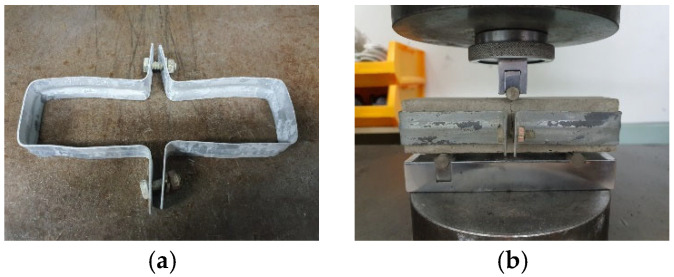
Flexural reloading test: (**a**) clamping device used to maintain specimen integrity after crack formation, (**b**) overview of the reloading test setup.

**Figure 6:**
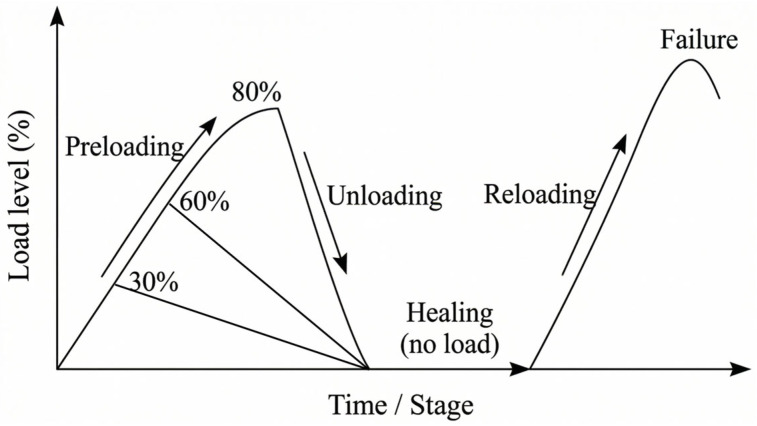
Schematic illustration of the loading, unloading, healing, and reloading procedure used in this study.

(d) Test setup and terminology: Compression reloading was conducted directly on preloaded prisms; flexural reloading used a clamping fixture and loading. Figure 5 presents the flexural reloading test setup using a clamping device to maintain specimen integrity after crack formation, while Figure 6 illustrates the loading–unloading–healing–reloading procedure applied to the preloaded specimens. The compressive load was reapplied directly to the preloaded specimens without any additional attachments. The clamping device was employed in flexural tests to maintain specimen integrity after crack formation during preloading and to ensure consistent load transfer during reloading.

A clamping device was used after crack initiation to maintain specimen integrity and control crack opening during the healing period, rather than to prevent crack formation.

This approach enabled stable reloading conditions without complete separation of the cracked specimen.

#### 2.3.4. Specimen Preparation and Test Configuration

For the experimental program, specimens of different types and sizes were prepared depending on the test method, and all specimen dimensions were selected in accordance with relevant testing standards.

For compressive strength, cylindrical specimens (Ø100 × 200 mm) were used, and three specimens were tested per mix. Flexural strength was evaluated using prismatic specimens (100 × 100 × 400 mm), with three specimens per mix.

For the chloride ion penetration test, cylindrical specimens (Ø100 × 200 mm) were sectioned into four slices, and two central slices were used as test specimens. A total of four specimens were tested per mix. For carbonation tests, cylindrical specimens (Ø100 × 200 mm) were cut in half, and three specimens were tested per mix. Freeze–thaw resistance was evaluated using prismatic specimens (100 × 100 × 400 mm), with three specimens per mix.

For mechanical healing evaluation, mortar specimens (40 × 40 × 160 mm) were used. In compressive strength recovery tests, specimens were split into two halves according to KS L ISO 679 [31], and four specimens were tested per mix. Flexural healing performance was evaluated using three specimens per mix.

The mechanical healing test was conducted as an initial exploratory study. Therefore, HC-0 and a representative intermediate replacement ratio (HC-3) were selected to evaluate healing performance while minimizing experimental complexity. Further studies will consider a wider range of replacement ratios to investigate the effect of capsule content on mechanical healing behavior.

## 3. Results and Discussion

### 3.1. Fundamental Properties

Table 4 summarizes the fundamental properties of concrete incorporating hybrid capsules (HC). The slump of the mixtures gradually decreased with increasing HC content, from approximately 175 mm for HC-0 to about 150 mm for HC-5, representing a 14% reduction. This reduction was mainly attributed to frictional loss and minor rupture of capsules during mixing, which may reduce the amount of free water available for flow. Previous studies [14,15,19] also reported that roughly 10% of the incorporated capsules can be damaged by aggregate friction and blade contact, leading to a comparable decline in workability. Despite this decrease, all mixes maintained slump values within the practical range for precast concrete, indicating that the inclusion of HC does not significantly impair workability.

The air content exhibited only a slight downward trend, decreasing from 3.3% (HC-0) to about 3.0% (HC-5), which falls within the experimental error range. The nearly constant air content suggests that the capsule addition did not significantly influence the entrapped or entrained air characteristics of the mixtures. Therefore, the influence of HC on fresh-state properties was limited primarily to a minor slump loss rather than any substantial change in air retention.

In terms of compressive strength, the 28-day values of HC-1, HC-3, and HC-5 were approximately 4%, 7%, and 16% lower than HC-0 (≈45.6 MPa). This reduction was primarily associated with the lower intrinsic strength of capsule particles and the presence of weak interfacial transition zones (ITZs) between the capsule surface and the cement matrix. However, the time-dependent strength development of all mixtures followed similar trends. This suggests that the overall hydration process was not significantly hindered of HC. This finding is consistent with the conclusions of Oh et al. (2019) [13] and Choi et al. (2022) [14], who reported that capsule contents up to 3% produce only minor reductions in ultimate strength without affecting the rate of strength gain.

The flexural strength also decreased as the HC content increased, from 9.6 MPa (HC-0) to 9.1 MPa (HC-5), corresponding to a reduction of approximately 5%. This slight reduction may be associated with the presence of ITZ around the capsules, which can act as local stress concentrators under bending. However, the increase in flexural strength from 7 days to 28 days was nearly parallel across all mixtures, indicating comparable strength development behavior regardless of HC content. This observation clarifies the previously ambiguous interpretation regarding strength development trends.

Overall, the inclusion of hybrid capsules slightly reduced the initial strength of the concrete due to the inert nature of the capsule particles but did not alter the rate of strength gain over time. These results suggest that hybrid capsules influence early-age load-bearing capacity while maintaining comparable long-term strength development, indicating their potential applicability in precast concrete systems without significantly compromising fundamental mechanical properties.

### 3.2. Durability Performance

Durability performance was examined only to ensure that the inclusion of hybrid capsules did not compromise the fundamental durability of the precast concrete. Representative tests, including chloride ion diffusion, carbonation, and freeze–thaw resistance, were conducted in accordance with KS F and ASTM standards [26,27,28,29,30]. The specific procedures followed standard accelerated methods under controlled temperature, humidity, and CO_2_ concentration conditions.

**Figure 7:**
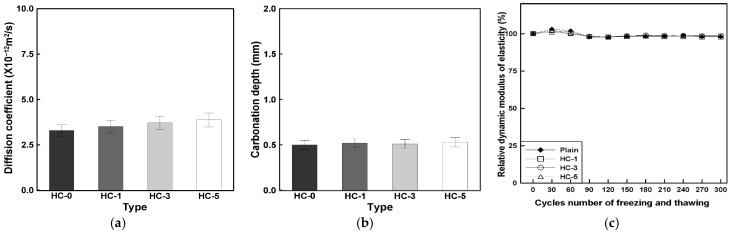
Durability performance evaluation results: (**a**) Chloride ion diffusion coefficient, (**b**) Carbonation depth, (**c**) Relative dynamic elastic modulus.

Figure 7 presents the results of the durability performance evaluation, including (a) chloride ion diffusion coefficient, (b) carbonation depth, and (c) relative dynamic elastic modulus after freeze–thaw cycles.

The chloride ion diffusion coefficient slightly increased with higher HC content, showing increments of approximately 7%, 13%, and 18% for HC-1, HC-3, and HC-5, respectively, compared to HC-0. However, all mixes recorded charge-passing values below 2000 C, classified as “Low” according to ASTM C1202 [32], indicating that chloride resistance was maintained within an acceptable range despite the observed increase.

The carbonation depth remained below 1 mm for all mixtures regardless of HC content, suggesting no notable change in carbonation resistance due to the inclusion of hybrid capsules. Similarly, after 300 freeze–thaw cycles, all specimens maintained more than 97% of their initial dynamic elastic modulus, indicating stable resistance to freeze–thaw action under the tested conditions.

Overall, the inclusion of hybrid capsules caused minor variations in individual durability indices but did not lead to any significant degradation. The results suggest that the hybrid capsule system can be incorporated without substantially compromising essential durability characteristics, thereby supporting its applicability as a functional additive while maintaining baseline durability performance. Therefore, the following section focuses on the mechanical healing behavior under load reapplication to further examine the functional role of hybrid capsules in terms of strength recovery after damage.

Although accelerated durability protocols (chloride migration, carbonation, and freeze–thaw) were conducted under controlled temperature, humidity, and CO_2_ concentration, such conditions may not fully replicate field environments, particularly for long-term moisture and ion transport. Hence, the present durability results should be interpreted as indicative of relative performance under controlled conditions rather than direct predictors of field behavior. Future work will couple accelerated and natural-exposure programs to derive transfer factors and acceptable limits tailored to specific precast applications.

### 3.3. The Mechanical Healing Performance Under Load Reapplication

#### 3.3.1. Compressive Strength Recovery 

**Figure 8:**
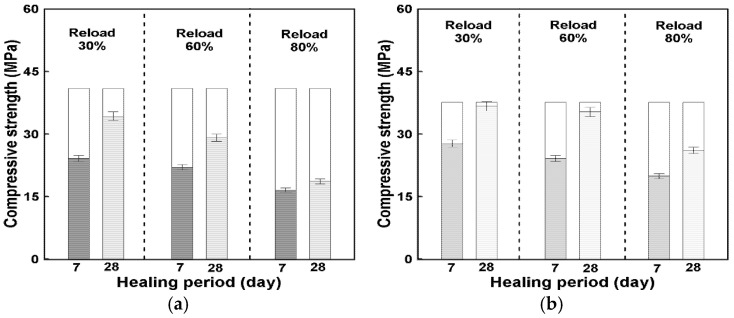
Relationship between healing period and reloading compressive strength according to preloading; (**a**) HC-0, (**b**) HC-3.

**Figure 9:**
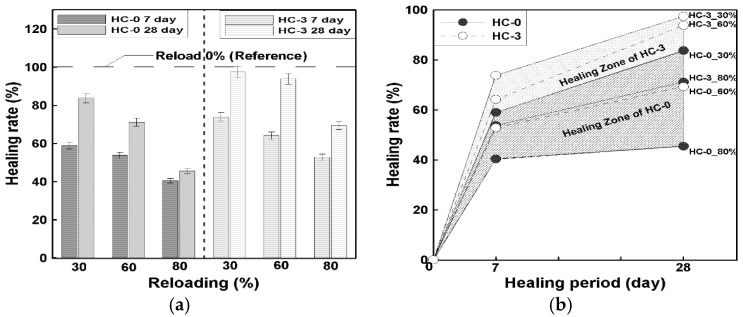
Relationship between healing period and compressive strength recovery according to preloading level; (**a**) Reloading based on healing age, (**b**) Effective healing zone according to HC mix ratio.

The mechanical healing performance of precast concrete incorporating hybrid capsules (HC) was evaluated through reloading tests under compressive and flexural strength conditions. Specimens were preloaded at 30%, 60%, and 80% of their 28-day failure strengths and then retested after healing periods of 7 and 28 days, as defined in Equation (2) in Section 2.3.3. The compressive strength recovery results are illustrated in Figure 8, and the corresponding healing rates are summarized in Figure 9.

It should be noted that the reference strength presented in this section is based on mortar-level specimens prepared without coarse aggregates for the healing test. Therefore, the reference compressive strength is lower than that of the original concrete mixture.

As shown in Figure 8, both HC-0 and HC-3 exhibited distinct strength recovery after the healing periods, but the specimens containing hybrid capsules demonstrated a higher level of relative recovery. At a 30% preloading level, which corresponds to the elastic range, the 7-day healing rate of HC-3 reached approximately 74%, compared with 59% for HC-0. After 28 days of healing, the recovery further increased to about 97% and 84%, respectively. This suggests that under relatively minor damage conditions, the presence of capsules may contribute to crack closure and strength recovery during the healing process.

At the 60% preloading level, representing partial inelastic damage, HC-3 achieved healing rates of 64% (7 days) and 94% (28 days), whereas HC-0 recorded 54% and 71%, respectively. The improvement of over 20 percentage points at 28 days indicates a notable difference in recovery behavior between the mixtures, which may be associated with the presence of healing agents within the capsules. In contrast, at the 80% preloading level—corresponding to near-failure damage—the healing effect was less pronounced but still measurable. HC-3 recovered approximately 69% of its strength after 28 days, compared with 46% for HC-0, demonstrating a relative gain of about 23%. It should be noted that the observed strength recovery may include combined effects of self-healing and continued hydration, as discussed in Section 2.3.3.

Figure 9a,b illustrate the overall healing trend and effective healing zones for each mixture. The parallel increase in healing rate over time for all preloading levels confirms that the recovery process was progressive and time-dependent. The distinct difference between HC-0 and HC-3 was most evident under severe damage conditions, where the presence of capsules may contribute to additional recovery beyond that observed in the reference mixture. These findings suggest that hybrid capsules may influence compressive strength recovery through mechanisms such as crack closure and possible secondary reactions within damaged regions.

Overall, the results indicate that the inclusion of hybrid capsules is associated with an increase in compressive strength recovery, with differences of approximately 10–20% compared to the control mix (HC-0), particularly under higher preloading conditions. This suggests the potential of the hybrid capsule system to enhance recovery behavior, although the results should be interpreted as relative improvements under the given experimental conditions rather than absolute performance gains.

#### 3.3.2. Flexural Strength Recovery

**Figure 10:**
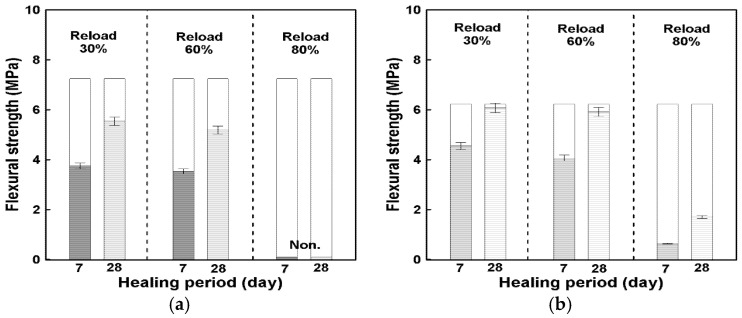
Relationship between healing period and reloading flexural strength according to preloading; (**a**) HC-0, (**b**) HC-3.

**Figure 11:**
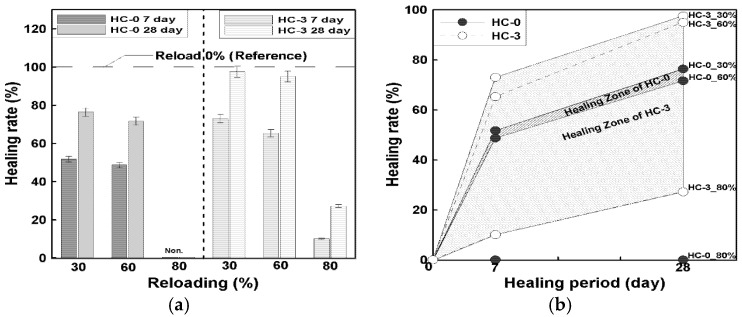
Relationship between healing period and flexural strength recovery according to preloading level; (**a**) Reloading based on healing age, (**b**) Effective healing zone according to HC mix ratio.

The flexural strength recovery of the specimens after load reapplication was evaluated using the same procedure described for the compressive tests, with healing rates calculated according to Equation (2) in Section 2.3.3. The experimental results are presented in Figure 10, while Figure 11 summarizes the corresponding healing rates and effective healing zones for HC-0 and HC-3.

Similarly, the reference flexural strength is based on specimens without coarse aggregates, and thus differs from the strength of the original concrete mixture.

As shown in Figure 10, both mixtures exhibited measurable flexural strength recovery after the healing periods, but the inclusion of hybrid capsules led to a higher level of relative recovery. At the 30% preloading level (elastic range), HC-3 achieved a healing rate of approximately 73% at 7 days and 98% after 28 days, whereas HC-0 recovered about 52% and 76%, respectively. This suggests that under relatively low-damage conditions, the presence of capsules may contribute to improved recovery behavior during the healing process.

At 60% preloading (partial inelastic range), the healing rate of HC-3 reached about 65% after 7 days and 95% after 28 days, which was approximately 23 percentage points higher than HC-0 (49% and 72%, respectively). This indicates a noticeable difference in recovery trends between the mixtures, which may be associated with the presence of healing agents within the capsules.

In contrast, at the 80% preloading level (nearly complete failure range), HC-0 showed no measurable healing, while HC-3 exhibited a clear recovery of about 10% at 7 days and 27% after 28 days. The results suggest that even under severe damage conditions, a certain level of recovery may occur in the presence of capsules, although the overall recovery remained limited.

During preloading, no visible surface cracks were observed in compressive specimens, whereas flexural specimens exhibited visible cracks at higher preloading levels (60% and 80%).

As depicted in Figure 11a, the difference between HC-0 and HC-3 became more pronounced as the preloading level increased. Figure 11b further illustrates the healing zones corresponding to different damage levels, where HC-3 maintained a consistently wider effective healing region across all preloading conditions. This indicates a broader range of recovery behavior associated with the hybrid capsule system under the tested conditions.

It should be noted that the healing rates were calculated based on the 28-day reference strength, and control specimens at equivalent ages (e.g., 35 and 56 days) were not included. Therefore, the observed recovery may include combined effects of self-healing and continued hydration, and should be interpreted as relative recovery rather than absolute healing performance.

Overall, the flexural tests indicate that the inclusion of hybrid capsules is associated with an increase in flexural strength recovery, with differences of approximately 20% compared with the control mix. These findings suggest the potential applicability of hybrid capsule concrete in improving recovery behavior under flexural loading, although the results should be interpreted within the limitations of the present experimental framework.

#### 3.3.3. Discussion on Healing Mechanism and Durability Correlation

**Figure 12:**
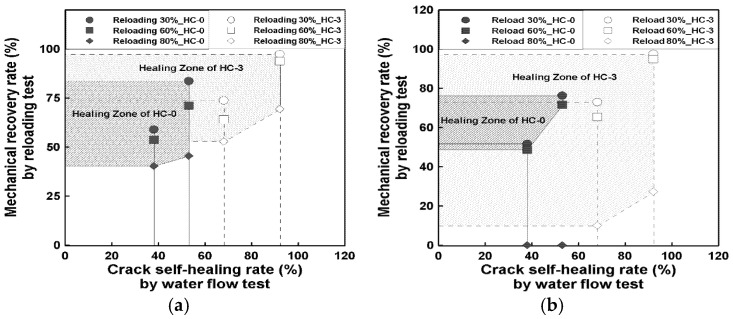
Correlation between water flow test and reloading test; (**a**) Compressive load, (**b**) Flexural load.

Figure 12 presents the relationship between the self-healing rate obtained from the water-flow test and the mechanical recovery rate measured by the reloading test under both compressive and flexural strength conditions. The water-flow data were referenced from a previous study [14,19], which employed constant-head permeability tests under similar healing environments. Since the datasets were not obtained from identical specimens, the comparison is intended as a conceptual reference rather than a direct quantitative correlation.

**Figure 13:**
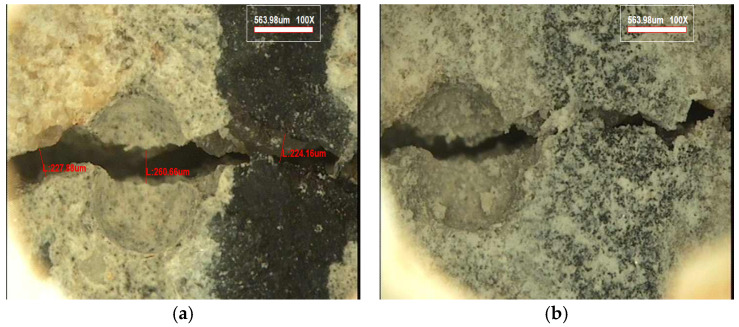
Microscopic images of crack surfaces in plain concrete (HC-0); (**a**) initial crack condition, (**b**) crack condition after 28 days of healing.

In Figure 12a, a general trend can be observed in which higher crack self-healing rates from the water-flow test correspond to higher mechanical recovery rates. At a 30% preloading level, both HC-0 and HC-3 exhibited moderate recovery behavior, with HC-3 showing a relatively higher recovery tendency compared to HC-0. At higher preloading levels (60% and 80%), the recovery region of HC-3 remained broader than that of HC-0, indicating a consistent trend of enhanced recovery behavior in the presence of capsules under the compared conditions. A similar tendency is observed in Figure 13b for flexural reloading, where higher water-flow healing rates were generally associated with higher mechanical recovery levels.

The observed relationship suggests a possible linkage between permeability-related healing behavior and mechanical recovery, although the underlying mechanisms were not directly investigated in this study. Such behavior may be associated with crack closure and changes in the local matrix condition around damaged regions, which could influence both transport properties and load-bearing capacity.

However, the present correlation analysis should be interpreted as qualitative and exploratory, since the water-flow data were sourced from previous studies and not obtained from the same experimental batch. Therefore, no direct quantitative relationship can be established in the current study. Future work should involve integrated experimental programs to evaluate both durability and mechanical recovery using identical specimens.

Overall, the results suggest that hybrid capsule systems may contribute to both permeability reduction and mechanical recovery, although further validation is required to establish a coupled relationship between durability and mechanical healing performance.

Although direct comparison with previous studies is limited due to the scarcity of research on precast concrete subjected to load reapplication, the results of this study are consistent with the general trend that hybrid capsule systems enhance crack healing performance. Unlike most existing studies focusing on mortar-scale specimens or durability-related indicators, this study provides experimental evidence of mechanical recovery under structural-level conditions.

Although a formal statistical error analysis was not conducted, all experiments were performed under controlled conditions following standardized test methods, and consistent trends were observed across the specimens. Therefore, the reliability of the results is considered to be sufficient within the scope of this study.

#### 3.3.4. Microscopic Observation of Crack Healing Behavior

Microscopic observations were conducted to qualitatively evaluate the crack healing behavior of concrete with and without hybrid capsules. Figure 13 shows the crack condition of plain concrete (HC-0) at the initial stage and after 28 days of healing. Although slight changes in crack morphology were observed, the crack remained largely unfilled, indicating limited natural healing under the given conditions.

**Figure 14:**
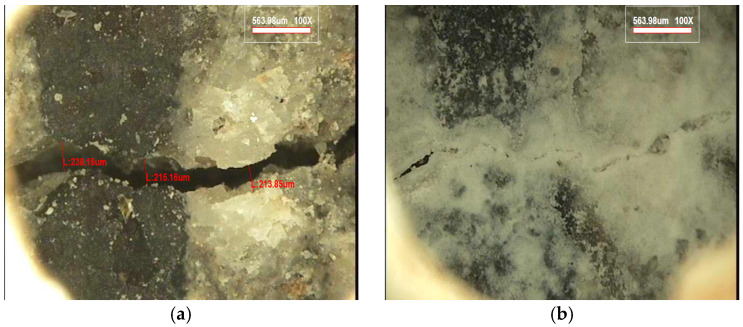
Microscopic images of crack surfaces in hybrid capsule–incorporated concrete (HC-3); (**a**) initial crack condition, (**b**) crack condition after 28 days of healing.

In contrast, Figure 14 presents the crack condition of hybrid capsule–incorporated concrete (HC-3). At the initial stage, clear crack openings similar to those observed in HC-0 were identified. However, after 28 days of healing, the crack region was significantly filled with healing products, resulting in a visibly reduced crack width and partial to near-complete closure.

These observations indicate that the incorporation of hybrid capsules effectively enhances crack filling compared to plain concrete. The healing products are considered to be generated through the activation of solid and liquid capsules, which provide complementary mechanisms for crack closure.

Overall, the microscopic results provide direct visual evidence supporting the enhanced healing performance of hybrid capsule systems and are consistent with the mechanical recovery trends observed in the reloading tests.

## 4. Conclusions

In this study, the applicability of precast concrete incorporating hybrid capsules (HC) as a smart construction material was investigated. The effects of hybrid capsules on the fundamental properties, durability, and mechanical healing performance of precast concrete were evaluated, leading to the following conclusions.

1. The inclusion of HC slightly reduced the slump of concrete by approximately 14%, from 175 mm (HC-0) to 150 mm (HC-5), but this reduction was not significant enough to impair workability. The air content remained nearly constant (3.3 ± 0.3%), indicating that HC had a limited influence on the fresh-state properties.
2. The compressive and flexural strengths at 28 days decreased by approximately 7% and 5%, respectively, compared to the control mix (HC-0), due to the lower intrinsic strength of capsule particles and the formation of weak interfacial transition zones (ITZs). Nevertheless, the rate of strength development over time was similar across all mixtures, suggesting that HC did not significantly hinder overall hydration or strength development trends.
3. The inclusion of HC caused only minor variations in durability indicators. The chloride ion diffusion coefficient slightly increased with HC content but remained within the “Low” range (below 2000 C per ASTM C1202). The carbonation depth was less than 1 mm, and all specimens maintained more than 97% of their initial dynamic elastic modulus after 300 freeze–thaw cycles. Therefore, HC can be considered to have a limited influence on durability performance within the tested conditions.
4. Mechanical reloading tests revealed that hybrid capsules were associated with enhanced relative recovery of compressive and flexural strengths, particularly under higher preloading levels. At 80% preloading, HC-3 exhibited approximately 69% compressive and 27% flexural recovery after 28 days, corresponding to about 23 and 11 percentage-point differences compared to HC-0, respectively. This suggests that the presence of hybrid capsules may contribute to improved recovery behavior after damage, although the observed recovery may include combined effects of self-healing and continued hydration.
5. The relationship between durability and mechanical healing performance showed a general positive trend, where higher self-healing rates from the water-flow test were associated with higher mechanical recovery levels. However, since the datasets were obtained from separate studies, this relationship should be interpreted as qualitative and exploratory rather than a direct quantitative correlation.

Overall, precast concrete incorporating hybrid capsules exhibits stable quality and maintains baseline durability performance while showing improved recovery behavior under the given experimental conditions. These findings suggest the feasibility of hybrid capsule systems as functional additives in precast concrete [33], although the results should be interpreted as relative performance within the experimental framework rather than absolute validation of self-healing efficiency.

In addition, the observed healing performance may be influenced by several parameters, including capsule distribution, crack geometry, and preloading level, which could introduce variability in the results. While consistent trends were observed across the tested conditions, these factors may affect the accuracy and reproducibility of the measured recovery behavior. Therefore, the results should be interpreted with consideration of these influencing parameters within the experimental scope.

Future studies should establish quantitative relationships between mechanical and durability healing indices and verify long-term performance under field conditions.

This study focuses on the feasibility of mechanical healing performance in precast concrete incorporating hybrid capsules. Further investigations, including extended experimental datasets, field-scale validation, and economic feasibility, are currently ongoing and will be reported in future studies.

## Figures and Tables

**Table 1:** Chemical composition of expansion materials [19].

Component	Typical Content (%)	Function in Healing Reaction
Calcium oxide (CaO)	45–55	Releases Ca^2+^ during hydration
Calcium sulfate (gypsum)	25–35	Supplies sulfate ions
Calcium sulfoaluminate	5–10	Promotes expansive crystal formation
errite phase (C_2_F + Fe_2_O_3_)	1–5	Minor auxiliary phase

**Table 2:** Chemical composition and physical properties of silicate-based materials.

Type	Potassium Silicates	Sodium Silicates	Lithium Silicates
Specific gravity (20 °C)	1.27–1.29	1.25–1.27	1.20–1.22
K_2_O (%)	10.0–11.0	–	–
Na_2_O (%)	–	9.0–10.0	–
Li_2_O (%)	–	–	8.0–9.0
SiO_2_ (%)	21.5–22.5	22.0–23.0	24.0–25.0
Fe_2_O_3_ (%)	0.05	0.03	–
Mole fraction	3.2–3.5	3.1–3.4	3.2–3.6
Viscosity (cps, 20 °C)	≤20	≤25	≤30
Solid content (%)	20–52	30–56	25–50

**Table 3:** Mix proportions of precast concrete [19].

Mix ID	W/B	S/a	Unit Mass (kg·m^3^)				Admixture (C Mass x %)	HC Replacement (S vol. x %)
			Water	Cement	Sand	Gravel		
HC-0	39.3	49.0	165	420	862	908	0.8	0
HC-1	39.3	49.0	165	420	862	908	0.8	1
HC-3	39.3	49.0	165	420	862	908	0.8	3
HC-5	39.3	49.0	165	420	862	908	0.8	5

**Table 4:** Fundamental properties of precast concrete incorporating hybrid capsules.

Mix Type	Flow (mm)			Air Content (%)	Compressive Strength (MPa)			Flexural Strength at 28 Days (MPa)
	0 min.	30 min.	60 min.		3 Days	7 Days	28 Days	
HC-0	175	150	133	3.3	30.5	35.0	45.6	9.6
HC-1	168	140	128	3.2	30.1	34.5	43.8	9.3
HC-3	162	137	121	3.1	29.6	33.9	42.4	9.2
HC-5	150	122	110	3.0	29.6	33.1	38.3	9.1

## Data Availability

The original contributions presented in the study are included in the article, further inquiries can be directed to the corresponding authors.
